# Clinical efficacy of early loading versus conventional loading of dental implants

**DOI:** 10.1038/srep15995

**Published:** 2015-11-06

**Authors:** Yanfei Zhu, Xinyi Zheng, Guanqi Zeng, Yi Xu, Xinhua Qu, Min Zhu, Eryi Lu

**Affiliations:** 1 College of Stomatology, Shanghai JiaoTong University School of Medicine, Shanghai, China; 2 Department of Orthopaedics, Shanghai Ninth People’s Hospital, Shanghai JiaoTong University School of Medicine, Shanghai Key Laboratory of Orthopaedic Implant, Shanghai, China; 3 Department of Oral and Cranio-Maxillofacial Science, Shanghai Ninth People’s Hospital, College of Stomatology, Shanghai Jiao Tong University School of Medicine, Shanghai Key Laboratory of Stomatology, Shanghai, China; 4 Department of Prosthodontics, Shanghai Ninth People’s Hospital, College of Stomatology, Shanghai JiaoTong University School of Medicine, Shanghai, China

## Abstract

The aim of this study was to determine the clinical differences between early and conventional loading protocols for dental implants. A comprehensive search of the Medline, Embase, and OVID databases for studies published through January 10, 2015 was conducted. Fourteen studies were included in our analysis. We found that early loading imposed a significantly higher risk of implant failure than did conventional loading (risk ratio = 2.09, 95% confidence interval [CI] [1.18, 3.69], P = 0.01), while no significant differences between the methods were found with regards to the marginal bone loss (weighted mean differences [WMD] = 0.11, 95% CI [−0.07, 0.28], P = 0.23), periotest value (WMD = 0.02, 95% CI [−0.83, 0.87], P = 0.96), or implant stability quotient (WMD = 0.79, 95% CI [−0.03, 1.62], P = 0.06). As for the health status of the peri-implant tissue, conventionally loaded implants demonstrated better performance than did early loaded implants. Subgroup analyses demonstrated that the sample size, time of publication, loading definition, implant position, extent, and restoration type influenced the results. Although early implant loading is convenient and comfortable for patients, this method still cannot achieve the same clinical outcomes as the conventional loading method.

Since Branemark introduced the osseointegration system in 1977^1^, a healing period of at least 3–4 months without loading has been advocated to achieve osseointegration of dental implants^2^. Any micromotion of the implant may disturb the healing process causing fibrous scar tissue to form that separates the implant and bone rather than promoting bone apposition^3^,^4^. When the micromotion reaches a certain threshold, it can eventually lead to failure of the implant^5^.

However, this nonloading period is usually troublesome and is associated with functional and aesthetic disturbances, especially in completely edentulous patients due to the need to use removable dentures^6^,^7^. With the evolution of dentistry over the last few decades, this nonloading period has become shorter. Branemark’s protocols have been reevaluated and modified significantly by the development of non-submerged healing for two-stage implants, immediate or early implant placement after tooth extraction, and immediate or early loading. Moreover, with the improvement of implant surface technology, which has shortened the loading waiting period from 12–24 weeks to 6–8 weeks without reducing the success rate, faster and steadier osseointegration can be attained^8^.

A recent meta-analysis corroborated the findings of previous studies showing that immediate loading is associated with a significantly higher implant failure rate, lower marginal bone loss, and a higher implant stability quotient (ISQ) compared to conventional loading^9^. However, to the best of our knowledge, no study has reported the clinical differences between early and conventional loading. Therefore, the aim of this study was to determine whether or not early loading resulted in different clinical outcomes than conventional loading in terms of the implant failure rate, marginal bone loss, implant stability, peri-implant parameters, and complications.

## Methods

### Focused question

We hoped to answer the following question by developing a protocol: does early implant loading produce different results than conventional loading in terms of the implant failure rate, marginal bone loss, implant stability, health of the peri-implant soft tissue, and complications. This question was developed by using the following PICOs definitions:

- Population: patients who need at least one implant, older than 18 years, having adequate oral hygiene, having adequate alveolar bone volume to place implants, and in good general health to permit implant surgery.
- Intervention: early loading of implants (test group) was defined as implants loaded between 1 week and 2 months after insertion.
- Comparison: conventional loading of implants (control group) was defined as implants loaded more than 2 months after insertion; also referred to as delayed loading^10^.
- Outcomes: (1) the primary outcome was the implant failure rate, defined as the rate of mobility and removal of implants^11^; and (2) the secondary outcomes were: (a) marginal bone loss (mm): measured as the distance from the implant shoulder to the first osseointegration of each implant by radiographic analysis; (b) implant stability: measured as the ISQ and periotest value (PTV); (c) peri-implant tissue health status, which included the relative attachment level (AL), probing pocket depth (PD), modified bleeding index (BI), modified gingival index (GI), and plaque index (PI); and (d) advent of complications (technical and biological): technical complications were defined as mechanical damage of the implant components including fractures and loosening of the screws or abutments, while biological complications were defined as events that directly concerned the implants, varying from numbness of the lower lip to peri-implant infection.
- Study design: randomized controlled trials, prospective clinical comparative studies and retrospective clinical comparative studies were included.

### Search strategies

An electronic search limited to English publications with abstracts was conducted using the Medline, Embase, and OVID databases, and included all studies published through January 10, 2015.

The following search terms and strategies were used: (dental implant OR oral implant) AND (early loading OR early restoration OR early prostheses OR early overdenture) AND (conventional loading OR conventional restoration OR conventional prostheses OR conventional overdenture) AND (delayed loading OR delayed restoration OR delayed prostheses OR delayed overdenture).

The reference lists of identified studies and relevant reviews on the same subject were also searched for other potential studies.

### Inclusion and exclusion criteria

The inclusion criteria were as follows: (1) human studies, (2) clinical comparative studies, (3) studies comparing the implant failure rates in any group of patients who received early and conventionally loaded implants, (4) studies providing data regarding the relevant clinical outcomes, (5) studies with follow-up periods of at least 6 months after loading, and (6) studies published in English.

Furthermore, we used the following exclusion criteria: (1) *in vitro* or laboratory studies, (2) animal studies, (3) reviews, (4) case reports, (5) craniofacial implant studies, and (6) studies only focusing on the different clinical efficacy between implants loaded after less than 1 week (immediately loaded implants) and implants loaded conventionally.

### Study selection

Two reviewers (Y. Zhu and Y. Xu) screened the titles and abstracts independently for relevant subjects. Studies that could not be excluded definitively based on the information from the abstracts were searched using the full manuscript to determine whether they should be included. If an agreement could not be reached regarding inclusion/exclusion between the two reviewers, a discussion was held with a third reviewer until an agreement was reached (X. Qu).

### Data extraction

Two reviewers (Y. Zhu and Y. Xu) used a specially designed extraction form to extract data independently. Any disagreement about the data extraction was discussed with a third reviewer (X. Qu). The percentage of agreement and Kappa analyses were calculated to evaluate the inter-reviewer reliability of data extraction. The most complete data were extracted when the results of a study were published more than once or presented in different publications. The following information was extracted: first author’s name, country, year of publication, study design, follow-up period, number and age of patients, loading protocol, number and placement position of implants (maxilla or mandibular), extent of restoration (full or single implant), type of restoration (fixed or removable), type of implant system, and data on dental implant failure, marginal bone loss, ISQs, PTVs, and peri-implant parameters (AL, PD, BI, GI, PI).

### Quality assessment

The recommendations by Cochrane^12^ were used to assess the risk of bias. Studies were classified as having low risk of bias if they met the following criteria: (1) random allocation, (2) clear selection criteria for subjects, (3) blinding of the patients and examiners, (4) detailed reporting of the outcome data, (5) selection of representative population groups, and (6) use of identical treatments between two groups except for the intervention. If a study missed one of these criteria, it was considered to have a moderate risk of bias. Missing two or more of these criteria led to a high risk of bias^13^.

### Data analysis

For dichotomous variables (implant failure rates), the risk ratio (RR) and 95% confidence interval [CI] were calculated to compare the selected studies. For continuous variables (marginal bone loss, ISQ, PTV, and peri-implant parameters), the weighted mean difference (WMD) and 95% CI were used.

Heterogeneity among studies was tested through Q-tests and I^2^ statistics (I^2^ ≤ 25%: low, 25% < I^2^ < 50%: moderate, I^2^ ≥ 75%: high)^14^.

A fixed-effects model was used as a common measure for study-specific estimates, while a random-effects model was considered if significant heterogeneity was found^15^. The level of statistically significant heterogeneity was set at P < 0.1. In addition, subgroup analyses were conducted whenever possible to identify associations between the risk of implant failure, marginal bone loss, and other relevant elements as possible sources of heterogeneity. The subgroup analyses used the following explanatory variables: (1) length of follow-up, (2) sample size (implants), (3) design of study, (4) geographical location, (5) time of publication, (6) definition of early loading, (7) definition of conventional loading, (8) extent of restoration, (9) implant placement position, and (10) type of restoration.

Visualization of funnel plots, Begg’s test, and Egger’s regression tests were measured to assess publication biases^16^. Forest plots were drawn to demonstrate the effects in the meta-analyses. Stata version 11 (StataCorp, College Station, TX) and Review Manager 5.2 (The Cochrane Collaboration, Copenhagen, Denmark) were used to conduct the statistical analyses.

## Results

### Literature Search

**Figure 1:**
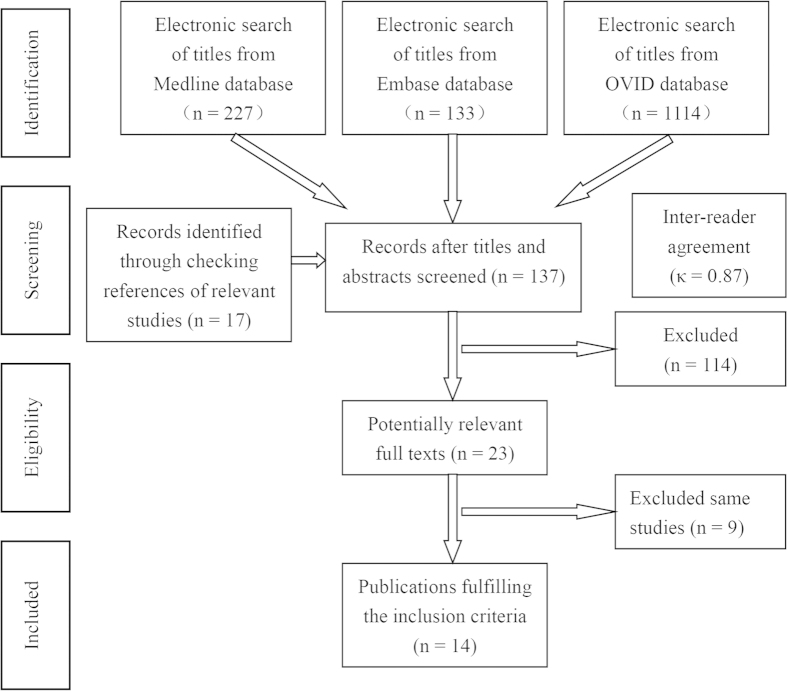
Flow-chart depicting the literature search procedure.

Figure 1 provides a flow chart of the literature search process. The search yielded a total of 1474 primary papers. Of these, 1360 papers were discarded by the two reviewers, leaving 114 papers after the titles and abstracts were evaluated (inter-rater agreement = 86%, kappa = 0.87). An additional 17 papers were included after searching the references of relevant studies, resulting in 137 papers that required full-text evaluation. After the full-text evaluation, 23 papers that reported data from 14 investigations remained. The data from four papers reporting on three investigations were repeated^17^,^18^,^19^,^20^,^21^,^22^,^23^ and five papers from two investigations reported different results on the same material in different years^24^,^25^,^26^,^27^,^28^,^29^,^30^. Therefore, nine papers were excluded, leaving 14 papers in the final analyses. One of the included papers was a three-armed study that utilized two experimental groups based on the different definitions of early loading^31^. Another of the included papers performed two controlled experiments depending on the different types of implant systems^32^. These two papers were divided into two parts when calculated.

### Study characteristics

The detailed study characteristics are presented in Table 1. In the 14 studies, 1619 implants were completely inserted, while 1265 implants remained after years of follow-up (681 implants in the test group, 584 implants in the control group). The number of implants in each study ranged from 20^33^ to 490^20^. The earliest study was published in 2000^34^, whereas the latest was published in 2012^30^. The follow-up period of the studies ranged from a minimum of 6 months^33^ to a maximum of 10 years^31^. As for the study design, six studies enrolled patients prospectively^18^,^33^,^34^,^35^,^36^,^37^, seven were randomized controlled trials (RCTs)^23^,^26^,^30^,^31^,^32^,^38^,^39^, and one was a retrospective study^20^. In terms of the geographic locations, four studies were conducted in Asia^23^,^30^,^33^,^37^, seven in Europe^18^,^20^,^31^,^34^,^35^,^36^,^39^, and three in Oceania^26^,^32^,^38^.

The group definitions and prosthetic characteristics are presented in Supplementary Table S1. In both groups, different definitions were used. Ten studies defined loading within 3 weeks as early loading^18^,^20^,^23^,^26^,^30^,^31^,^33^,^34^,^35^,^36^, while the other five studies placed the prostheses within 6 weeks^31^,^32^,^37^,^38^,^39^. One study had two experimental groups: one group had the implants loaded within 3 weeks and the other group had the implants loaded within 6 weeks^31^. The majority of studies in the conventional group loaded implants 3–4 months after insertion, except one, which loaded the implant after 6 months^37^.

Two studies inserted implants in the maxilla^26^,^37^, while 11 studies inserted implants in the mandible^18^,^20^,^23^,^30^,^31^,^32^,^33^,^34^,^35^,^36^,^38^. One study placed implants both in the maxilla and in the mandible^39^. For completely edentulous patients, overdentures were used in eight studies^23^,^30^,^31^,^32^,^33^,^35^,^36^,^38^ and fixed full-arch dental prostheses were used in four studies^18^,^20^,^26^,^34^. Fixed single implants were used in two studies^37^,^39^.

## Study Outcomes

### Implant failure

**Figure 2:**
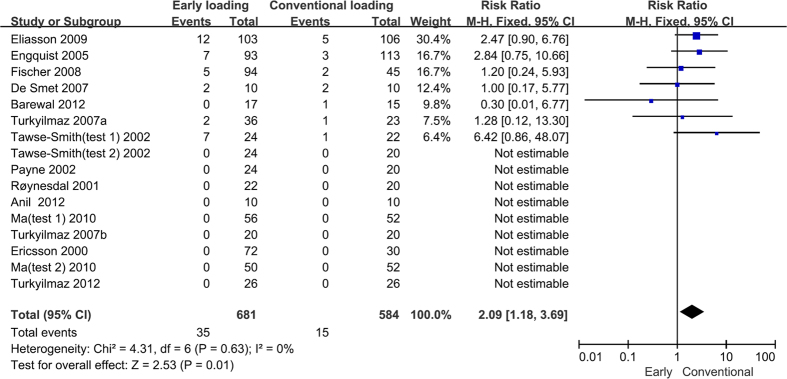
Forrest plots of the individual studies for the implant failure rate. Note that Tawse-Smith (2002) (test 1) and (test 2) indicate that in the same study, two controlled experiments were performed. Ma (2010) (test 1) and (test 2) indicate that in the same study, two different test groups were compared to the same control group.

In seven of the 14 included studies, no implant failure occurred in the study groups^23^,^30^,^31^,^33^,^34^,^35^,^38^. In all of the studies except one^39^, the reported implant failure rate was higher in the early loading group (0–29.2%) than it was in the conventional loading group (0–20%). The results showed that early loading imposed a significantly higher risk of implant failure compared to conventional loading (RR = 2.09, 95% CI [1.18, 3.69], P = 0.01). There was low heterogeneity among the included studies (P = 0.63, I^2^ = 0%). The results from the meta-analysis are presented in Fig. 2.

The subgroup analyses showed a higher risk of implant failure for early loaded implants restored with fixed prostheses (RR = 1.91, 95% CI [1.00, 3.64], P = 0.05) compared to conventionally loaded implants restored with fixed prostheses and for implants in the mandibular region (RR = 2.67, 95% CI [1.36, 5.24], P = 0.004) compared to implants in the maxilla . Patients who were over 50 years old (RR = 2.13, 95% CI [1.08, 4.19], P = 0.03) compared to those who were less than 50 and patients who were completely edentulous (RR = 2.37, 95% CI [1.28, 4.39], P = 0.006) compared to those who were singly edentulous had a higher risk of implant failure in the early loading groups. Similar outcomes occurred in the studies published before 2007 (RR = 2.57, 95% CI [1.11, 5.94], P = 0.03) compared to the studies published after 2007, in the studies that defined early implant loading as implants loaded 6 weeks after placement (RR = 2.60, 95% CI [1.17, 5.79], P = 0.02) compared to implants loaded 1 week or 3 weeks after placement, and in the studies that defined conventional implant loading as implants loaded less than 3 months after placement (RR = 2.78, 95% CI [1.07, 7.22], P = 0.04) compared to implants loaded more than 3 months after placement. The results from the subgroup analyses are presented in Table 2.

### Marginal bone loss

The marginal bone level in the early loading group ranged from 0.17 to 2.55 mm at baseline, from 0.97 to 2.87 mm at 1 year after loading, from 1.25 to 2.86 mm at 3 years, and from 1.61 to 2.98 mm at 5 years after loading; in the conventional loading group, the marginal bone level ranged from 0.11 to 3.5 mm at baseline, from 0.90 to 3.30 mm at 1 year after loading, from 1.26 to 3.5 mm at 3 years, and from 1.57 to 3.70 mm at 5 years after loading.

**Figure 3:**
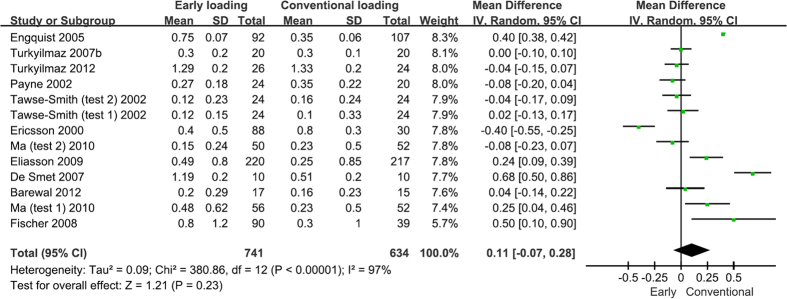
Forrest plots of the individual studies for the marginal bone loss.

The results of the meta-analysis regarding the overall marginal bone loss are presented in Fig. 3. Eleven studies that compared early and conventionally loaded implants reported marginal bone loss. The results illustrated that no significant difference was found in the overall marginal bone loss between these two loading protocols (WMD = 0.11, 95% CI [−0.07, 0.28], P = 0.23). Seven studies included marginal bone loss at 1 year after loading^18^,^20^,^23^,^31^,^32^,^36^,^38^. The results illustrated that early loaded implants had more marginal bone loss than did conventionally loaded implants after 1 year of loading (WMD = 0.18, 95% CI [0.00, 0.35], P = 0.05). Five studies included marginal bone loss at 2–3 years after loading^30^,^31^,^34^,^36^,^39^. No significant difference was found between the two loading types after 2–3 years of loading (WMD = 0.26, 95% CI [−0.15, 0.68], P = 0.21). Three studies reported the marginal bone loss at over 5 years after loading^26^,^30^,^31^. Again, no significant difference was found between the two groups after over 5 years of loading (WMD = 0.10, 95% CI [−0.10, 0.29], P = 0.33).

Subgroup analyses showed that the early loaded implants in the maxilla had a higher risk of marginal bone loss than did the implants in the mandibular region, while other elements had little significant influence on the marginal bone loss. The results from the subgroup analyses and meta-analysis on cumulative marginal bone loss are presented in Table 3.

### Implant stability

Clinical implant stability was measured by two methods: the Ostellt device, which measured the resonance frequency of a small transductor that was attached to the implant abutment and that was stimulated using a wide range of frequencies (ISQ); and the Periotest device, which measured the temporal contact of the tip of the instrument during repetitive percussions on the implant (PTV)^40^. Two studies were excluded since they only reported medians and ranges^35^,^38^. Therefore, PTVs were collected from four studies^32^,^33^,^34^,^36^. The mean values varied from −4.9 to −1.1 in the early loading group and from −4.52 to −3 in the conventional group. No statistically significant difference was found between the two groups (WMD = 0.02, 95% CI [−0.83, 0.87], P = 0.96).

**Figure 4:**
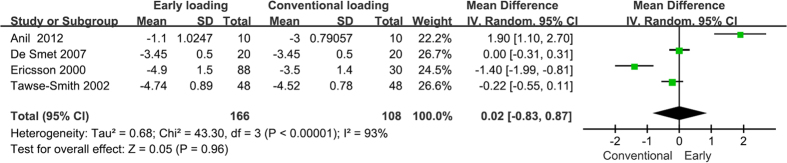
Forrest plots of the individual studies for the periotest value.

**Figure 5:**
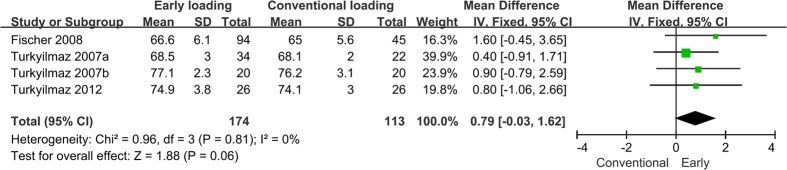
Forrest plots of the individual studies for the implant stability quotient.

In the four studies that reported ISQs^23^,^26^,^30^,^37^, the mean values varied from 66.6 to 81.9 in the test group and from 65 to 81.2 in the control group. Two studies were excluded because they lacked standard deviations^38^,^39^. The results showed a greater change in ISQ in the test group compared to in the control group, but this difference was not statistically significant (WMD = 0.79, 95% CI [−0.03, 1.62], P = 0.06). The forest plots of the PTVs and ISQs are presented in Figs 4 and 5 respectively.

### Peri-implant tissue health status

The peri-implant parameters were obtained from three studies^30^,^32^,^38^. Five different indexes were recorded to estimate the peri-implant tissue health status: AL, PD, BI, GI, and PI. The results showed that conventionally loaded implants had a better peri-implant tissue health status than did the early loaded implants in terms of their AL (WMD = 0.43, 95% CI [0.01, 0.85], P = 0.04) and PD (WMD = 0.26, 95% CI [0.11, 0.45], P = 0.001), while no statistically significant differences were observed between their BI (WMD = 0.12, 95% CI [−0.04, 0.29], P = 0.14), GI (WMD = 0.05, 95% CI [−0.11, 0.22], P = 0.52), and PI (WMD = 0.07, 95% CI [−0.17, 0.31], P = 0.58). However, there was an increase in these outcomes in the test group. The forest plots of these results are presented in Supplementary Figures S1–S5.

### Complications

Four studies reported technical complications^20^,^23^,^26^,^37^. The most common complications were fractures of the prostheses and loosening of the abutment screws. Technical complications occurred more often in the test groups according to the records from the included studies. All of these complications were solved by adjusting the implants or prostheses without affecting the outcomes. One study noted that denture contouring was the most common adjustment. The number of patients needing denture contouring adjustments was higher in the test group(15 people) than it was in the control group (12 people). This could be explained by the secondary gingival healing after surgery in the early loading group, which may result in space around the abutments, while relining impressions performed in the conventional loading group after a period of healing avoided this space^23^.

Biological complications were reported in three studies^20^,^26^,^33^. The most common complication was peri-implant inflammation, which eventually returned to normal.

Two studies reported no complications in either group^32^,^34^. None of the remaining studies mentioned any complications.

### Quality assessments

The results of the quality assessments of the included studies are presented in Supplementary Table S2. Of the 14 studies, one was considered to have a low risk of bias^39^, while three had a moderate risk of bias^31^,^32^,^38^. The remainder of the studies had a high risk of bias.

### Publication Bias

Publication bias was determined by Begg’s test, Egger’s regression test, and funnel plot visualization. With the exception of marginal bone loss (Begg’s test [B]: P = 0.093; Egger’s test [E]: P = 0.007), no evidence of publication bias was found for the failure rate (B: 0.453; E: 0.411), PTV (B: 1.00; E: 0.779), ISQ (B: 0.174; E: 0.087), PD (B: 0.117; E: 0.115), AL (B: 0.317), PI (B: 0.117; E: 0.042), GI (B: 0.317), or BI (B: 0.317). Given the limited number of included studies, only the publication bias of the implant failure rate was determined by funnel plot visualization. The funnel plot is presented in Supplementary Figure S6.

## Discussion

Immediate loading, early loading and conventional loading are the three main loading protocols in implantology. A recent meta-analysis has already comprehensively compared immediately loaded implants to conventionally loaded implants^9^. The results from that study demonstrated that immediate loading may impose a higher risk for implant failure compared to conventional loading. While early loading protocols (implants loaded 1 week to 2 months after insertion) permit a longer healing time than immediate loading protocols (implants loaded less than 1 week after insertion) and the efficacy difference between early loading and conventional loading protocols is uncertain, so the present meta-analysis, which utilized 14 different studies that compared early vs. conventional loading protocols with data from 1619 implants, aimed to establish whether early loading can achieve the same clinical outcomes as conventional loading with shorter time.

The results of this meta-analysis indicate that early loading has a higher risk of failure compared with conventional loading, which is not totally in agreement with the conclusions published recently in similar systematic reviews. The results from two recent meta-analyses demonstrated that the data were insufficient to determine whether or not there was a clinically important difference in implant failure or marginal bone loss between the two groups based on the limited number of available studies^10^,^41^. The results from two other meta-analyses did not show any significant differences between early and delayed loading^42^,^43^.

These discrepancies can be explained by the relatively small number of available studies together with their small sample sizes, which are not sufficient to establish definitive conclusions. Moreover, studies with a follow-up period of 4–12 months were included in a recent Cochrane systematic review, which was considered as not long enough to assess implant failure^10^. Therefore, the present meta-analysis included studies with a follow-up period of 6 months to 7 years.

Although recent systematic reviews have also studied the different failure rates between early and conventional loading^10^,^41^,^42^,^43^, the limitation of these studies was that insufficient data were available to perform comparative analyses of prosthodontic and peri-implant outcomes with reliable clinical results. In the present study, we enlarged the search scale and updated the search time frame. Thus, to our knowledge, the present study is the most comprehensive and newest meta-analysis estimating the clinical difference between early and conventional loading protocols in terms of implant failure, marginal bone loss, ISQ, PTV, and peri-implant parameters. The possible influence of the outcomes was also assessed through subgroup analyses involving the length of follow-up, sample size (implants), design of the study, geographical location, time of publication, definition of early loading, definition of conventional loading, extent of restoration, implant placement position, and type of restoration. The current meta-analysis also calculated the cumulative marginal bone loss in years, which gave a continuous view of the differences these two loading protocols caused over time, while other recent systematic reviews only presented the results of implant failure and marginal bone loss^10^,^41^,^42^,^43^.

Despite the strengths above, this meta-analysis still has several limitations. First, it was difficult to completely eliminate the confounding factors inherent in the included studies that may have resulted in a bias to the outcomes^20^. Second, the I^2^ estimates of marginal bone loss (I^2^ = 97%; P < 0.001) and PTV (I^2^ = 93%, P < 0.001) were evaluated as high. Heterogeneity introduced by methodological differences between the included studies could not be avoided. Therefore, subgroup analyses were conducted on studies of marginal bone loss, which indicated that the selected elements had no influence on the outcomes. The data on the PTV, however, were insufficient for a subgroup analysis. Third, the literature search was limited to studies in English and only covered three electronic databases, which might have resulted in an election bias to the outcomes. Considering the limitations mentioned above, the results of this meta-analysis should be interpreted with caution.

## Conclusions

The primary outcome of this study was that early loading had a higher risk of implant failure than did conventional loading. The secondary outcomes revealed that the two loading protocols were not significantly different in terms of the associated marginal bone loss, changes in implant stability, and health status of the peri-implant tissues (except for the AL and PD), which indicated that these two loading protocols behaved similarly once osseointegration occurred. Although early implant loading is advantageous with regards to convenience and comfort for patients, this method still cannot achieve the same clinical outcomes as the conventional loading. Careful selection of patients and surgery indications is still required. These findings may provide dentists with additional insights when developing treatment strategies and making prognoses. Meanwhile, based on this review, more high-quality and well-designed RCTs with large sample sizes are needed.

## Additional Information

**How to cite this article**: Zhu, Y. *et al.* Clinical efficacy of early loading versus conventional loading of dental implants. *Sci. Rep.* **5**, 15995; doi: 10.1038/srep15995 (2015).

## Supplementary Material

Supplementary Information

## Figures and Tables

**Table 1:** Study characteristics.

Author (Year)	Country	Study design	Follow-up	Mean age /range	Patients (Test/Control)	Implants (Test/Control)
Anil (2012)	India	Prospective clinical comparative study	6 months	45 ~ 65	5/5	10/10
Barewal (2012)	Poland	RCT	3 years	20 ~ 82	17/17	15/15
De Smet (2007)	Belgium	Prospective clinical comparative study	2 years	33 ~ 86	10/10	20/20
Ericsson (2000)	Sweden	Prospective clinical comparative study	5 years	66.3/53 ~ 77	16/11	88/30
Engquist (2005)	Sweden	Prospective clinical comparative study	3 years	64.9	26/30	108/120
Eliasson (2009)	Sweden	Retrospective clinical comparative study	5 years	47 ~ 90	55/54	248/242
Fischer (2008)	Sweden	RCT	5 years	39 ~ 79	16/8	95/47
Ma (2010) (test 1)	New Zealand	RCT	10 years	65	34/36	68/72
(test 2)					36/36	72/72
Payne (2002)	New Zealand	RCT	2 years	55 ~ 80	12/12	24/24
Røynesdal (2001)	Norway	Prospective clinical comparative study	2 years	75.7/61 ~ 85	11/10	22/20
Tawse-Smith (2002)	New Zealand	RCT	2 years	55 ~ 80	24/24	48/48
Turkyilmaz (2007a)	Turkey	Prospective clinical comparative study	4 years	40/20 ~ 60	19/10	36/23
Turkyilmaz (2007b)	Turkey	RCT	2 years	62	10/10	20/20
Turkyilmaz (2012)	Turkey	RCT	7 years	63	13/13	26/26

RCT = randomized controlled trial.

Ma (2010) (test 1) and (test 2) mean that in the same study, two different test groups were compared to the same control group.

**Table 2:** Subgroup analysis to investigate differences between studies included in meta-analysis for failure rate.

Analyses	Subgroup	Number of studies	RR [95% CI]	P value	I^2^ (%)	P value for heterogeneity
Overall		14	2.09 [1.18, 3.69]	0.01	0%	0.63
Sample size (implant)	≤50	7	1.98 [0.68, 5.73]	0.21	40%	0.19
	>50	7	2.13 [1.08, 4.19]	0.03	0%	0.82
Time of publication	Before 2007	8	2.57 [1.11, 5.94]	0.03	0%	0.52
	After 2007	6	1.72 [0.78, 3.78]	0.18	0%	0.39
Definition of early	1 week	4	1.00 [0.17, 5.77]	1.00	/	/
	3 weeks	4	2.60 [1.17, 5.79]	0.02	0%	0.87
	6 weeks	4	2.72 [0.69, 10.77]	0.15	62%	0.11
Definition of conventional	≤3 months	8	2.78 [1.07, 7.22]	0.04	24%	0.27
	>3 months	5	1.93 [0.86, 4.33]	0.11	0%	0.64
Extent of restoration	Full-arch	12	2.37 [1.28, 4.39]	0.006	0%	0.62
	Single	2	0.72 [0.12, 4.22]	0.72	0%	0.46
Position of implants	Maxilla	2	1.22 [0.33, 4.58]	0.77	0%	0.96
	Mandibular	11	2.67 [1.36, 5.24]	0.004	0%	0.58
Type of restoration	Fixed	6	1.91 [1.00, 3.64]	0.05	0%	0.66
	Removable	8	2.86 [0.83, 9.86]	0.10	50%	0.16

**Table 3:** Subgroup analysis to investigate differences between studies included in meta-analysis for MBL (mm).

Analyses	Subgroup	Number of Studies	WMD [95% CI]	P value	I^2^ (%)	P value for heterogeneity
Overall		11	0.11 [−0.07, 0.28]	0.23	97%	<0.01
≤1 year		7	0.18 [−0.00, 0.35]	0.05	98%	<0.01
2 ~ 3 years		5	0.26 [−0.15, 0.68]	0.21	96%	<0.01
≥5 years		3	0.10 [−0.10, 0.29]	0.33	77%	<0.01
Length of follow-up	≤1 years	5	0.09 [−0.13, 0.32]	0.42	97%	<0.01
	2 ~ 3 years	3	0.11 [−0.52, 0.74]	0.74	98%	<0.01
	≥5 years	3	0.10 [−0.10, 0.29]	0.33	77%	<0.01
Sample size (implant)	≤50	5	0.10 [−0.09, 0.29]	0.31	91%	<0.01
	>50	6	0.11 [−0.15, 0.37]	0.39	97%	<0.01
Design of study	RCT	7	0.01 [−0.06, 0.07]	0.87	47%	0.06
	Prospective study	3	0.23 [−0.29, 0.74]	0.39	98%	<0.01
Geographical location	Asia	2	−0.02 [−0.09, 0.06]	0.64	0%	0.60
	Europe	6	0.23 [−0.05, 0.52]	0.1	96%	<0.01
	Oceania	3	−0.01 [−0.10, 0.09]	0.89	51%	0.09
Time of publication	Before 2007	6	0.08 [−0.17, 0.33]	0.52	98%	<0.01
	After 2007	5	0.11 [−0.03, 0.25]	0.23	97%	<0.01
Definition of early	1 week	3	0.21 [−0.16, 0.57]	0.27	96%	<0.01
	3 week	3	0.08 [−0.39, 0.55]	0.73	98%	<0.01
	6 week	4	−0.04 [−0.10, 0.03]	0.26	0%	0.72
Definition of conventional	≤3 months	7	0.08 [−0.11, 0.27]	0.41	96%	<0.01
	>3 months	3	0.09 [−0.43, 0.61]	0.73	98%	<0.01
Extent of restoration	Full-arch	10	0.11 [−0.07, 0.29]	0.23	97%	<0.01
	Single	1	0.04 [−0.14, 0.22]	0.66	/	/
Position of implants	Maxilla	1	0.50 [0.10, 0.90]	0.01	/	/
	Mandibular	9	0.09 [−0.10, 0.27]	0.37	97%	<0.01
Type of restoration	Fixed	5	0.14 [−0.19, 0.48]	0.4	97%	<0.01
	Removable	6	0.08 [−0.07, 0.23]	0.28	89%	<0.01

MBL = marginal bone loss.
