# Supplementary material for: Clinical efficacy of early loading versus conventional loading of dental implants
Source: Sci Rep. 2015 Nov 6;5:15995. doi: 10.1038/srep15995 (PMC4635353; doi:10.1038/srep15995)
Supplement: Supplementary Information [file srep15995-s1.pdf]

# Clinical efficacy of early loading versus conventional loading of dental implants

Yanfei Zhu[1#], Xinyi Zheng[1#], Guanqi Zeng[1], Yi Xu[1], Xinhua Qu[2*], Min Zhu[3*], Eryi Lu[4*]

[1]College of Stomatology, Shanghai JiaoTong University School of Medicine, Shanghai, China; [2]Department of Orthopaedics, Shanghai Ninth People's Hospital, Shanghai JiaoTong University School of Medicine, Shanghai Key Laboratory of Orthopaedic Implant, Shanghai, China; [3]Department of Oral and Cranio-Maxillofacial Science, Shanghai Ninth People's Hospital, College of Stomatology, Shanghai Jiao Tong University School of Medicine, Shanghai Key Laboratory of Stomatology, Shanghai, China; [4]Department of Prosthodontics, Shanghai Ninth People's Hospital, College of Stomatology, Shanghai JiaoTong University School of Medicine, Shanghai, China.

#Yanfei Zhu and Xinyi Zheng contributed equally to this study.

*Corresponding author: xinhua_qu@126.com (X. Qu); zminnie@126.com (M. Zhu) ; lueryi222@126.com (E. Lu)

# Supplementary information

| Study or Subgroup | Early loading Mean | SD | Total | Conventional loading Mean | SD | Total | Weight | Mean Difference IV, Fixed, 95% CI |
|---|---|---|---|---|---|---|---|---|
| Payne 2002 | 2.07 | 0.21 | 24 | 1.78 | 0.5 | 20 | 45.3% | 0.29 [0.06, 0.52] |
| Tawse-Smith 2002 | 1.91 | 0.44 | 24 | 1.68 | 0.68 | 22 | 22.3% | 0.23 [-0.10, 0.56] |
| Turkyilmaz 2012 | 1.48 | 0.4 | 26 | 1.23 | 0.6 | 26 | 32.4% | 0.25 [-0.03, 0.53] |
| **Total (95% CI)** | | | **74** | | | **68** | **100.0%** | **0.26 [0.11, 0.42]** |

Heterogeneity: Chi² = 0.10, df = 2 (P = 0.95); I² = 0%
Test for overall effect: Z = 3.27 (P = 0.001)

Mean Difference IV, Fixed, 95% CI

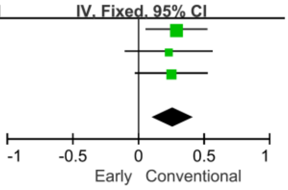


Figure S1. Forrest plots of the individual studies for probing pocket index.

| Study or Subgroup | Early loading Mean | SD | Total | Conventional loading Mean | SD | Total | Weight | Mean Difference IV, Fixed, 95% CI |
|---|---|---|---|---|---|---|---|---|
| Payne 2002 | 1.52 | 0.36 | 24 | 1.16 | 1.08 | 20 | 70.6% | 0.36 [-0.13, 0.85] |
| Tawse-Smith 2002 | 4.76 | 1.12 | 24 | 4.16 | 1.49 | 22 | 29.4% | 0.60 [-0.17, 1.37] |
| **Total (95% CI)** | | | **48** | | | **42** | **100.0%** | **0.43 [0.01, 0.85]** |

Heterogeneity: Chi² = 0.27, df = 1 (P = 0.61); I² = 0%
Test for overall effect: Z = 2.03 (P = 0.04)

Mean Difference IV, Fixed, 95% CI

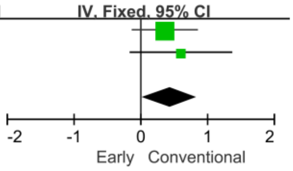


Figure S2. Forrest plots of the individual studies for the attachment level.

| Study or Subgroup | Early loading Mean | SD | Total | Conventional loading Mean | SD | Total | Weight | Mean Difference IV, Fixed, 95% CI |
|---|---|---|---|---|---|---|---|---|
| Payne 2002 | 0.63 | 0.76 | 24 | 0.6 | 0.78 | 20 | 27.1% | 0.03 [-0.43, 0.49] |
| Tawse-Smith 2002 | 1.17 | 0.79 | 24 | 1.13 | 0.73 | 22 | 29.4% | 0.04 [-0.40, 0.48] |
| Turkyilmaz 2012 | 0.89 | 0.7 | 26 | 0.78 | 0.6 | 24 | 43.6% | 0.11 [-0.25, 0.47] |
| **Total (95% CI)** | | | **74** | | | **66** | **100.0%** | **0.07 [-0.17, 0.31]** |

Heterogeneity: Chi² = 0.09, df = 2 (P = 0.95); I² = 0%
Test for overall effect: Z = 0.56 (P = 0.58)

Mean Difference IV, Fixed, 95% CI

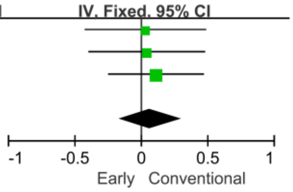


Figure S3. Forrest plots of the individual studies for the plaque index.

| Study or Subgroup | Early loading Mean | SD | Total | Conventional loading Mean | SD | Total | Weight | Mean Difference IV, Fixed, 95% CI |
|---|---|---|---|---|---|---|---|---|
| Payne 2002 | 0.28 | 0.36 | 24 | 0.23 | 0.33 | 20 | 63.0% | 0.05 [-0.15, 0.25] |
| Turkyilmaz 2012 | 0.92 | 0.3 | 26 | 0.86 | 0.6 | 24 | 37.0% | 0.06 [-0.21, 0.33] |
| **Total (95% CI)** | | | **50** | | | **44** | **100.0%** | **0.05 [-0.11, 0.22]** |

Heterogeneity: Chi² = 0.00, df = 1 (P = 0.95); I² = 0%
Test for overall effect: Z = 0.65 (P = 0.52)

Mean Difference IV, Fixed, 95% CI

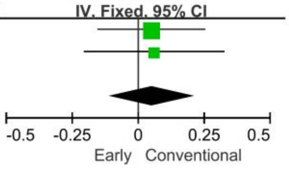


Figure S4. Forrest plots of the individual studies for the modified gingival index.

| Study or Subgroup | Early loading Mean | SD | Total | Conventional loading Mean | SD | Total | Weight | Mean Difference IV, Fixed, 95% CI |
|---|---|---|---|---|---|---|---|---|
| Tawse-Smith 2002 | 0.59 | 0.36 | 24 | 0.42 | 0.39 | 22 | 56.9% | 0.17 [-0.05, 0.39] |
| Turkyilmaz 2012 | 0.5 | 0.5 | 26 | 0.44 | 0.4 | 24 | 43.1% | 0.06 [-0.19, 0.31] |
| **Total (95% CI)** | | | **50** | | | **46** | **100.0%** | **0.12 [-0.04, 0.29]** |

Heterogeneity: Chi² = 0.42, df = 1 (P = 0.52); I² = 0%
Test for overall effect: Z = 1.46 (P = 0.14)

Mean Difference IV, Fixed, 95% CI

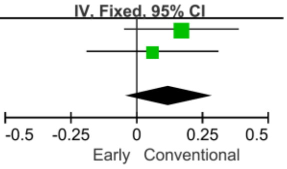


Figure S5. Forrest plots of the individual studies for the modified bleeding index.

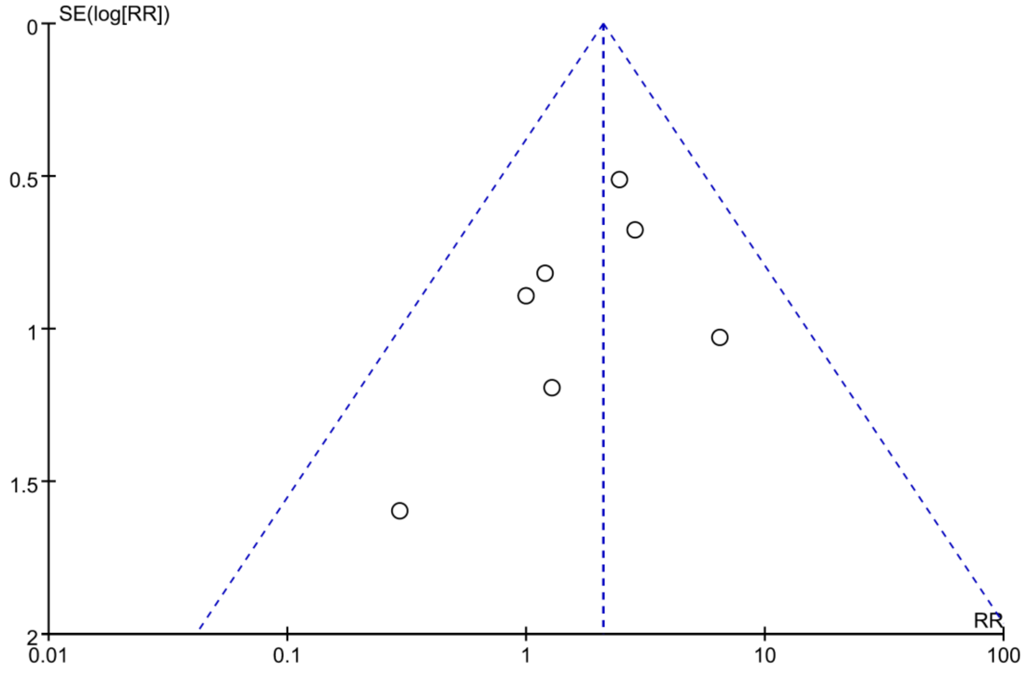


Figure S6. Funnel plot of the implant failure rates.

| Table S1. Definition of group and prosthetic characteristics | | | | | | |
|---|---|---|---|---|---|---|
| References | Definition-test | Definition-control | Extent of restoration | Position of implants | Type of restoration | Type of implants system |
| Anil (2012) | 1 week | 4 months | Full-arch | Mandible | Removable | Biohorizon Maestro |
| Barewal (2012) | 6 weeks | 3 months | Single | Mixed | Fixed | Astra Tech |
| De Smet (2007) | 1 week | 3~4 months | Full-arch | Mandible | Removable | Brånemark Novum |
| Ericsson (2000) | 20 days | 4 months | Full-arch | Mandible | Fixed | Brånemark MKII |
| Engquist(2005) | 3 weeks | 3 months | Full-arch | Mandible | Fixed | Brånemark MKIII |
| Eliasson (2009) | 3 weeks | 3 months | Full-arch | Mandible | Fixed | Brånemark, ITI, Astra Tech |
| Fischer (2008) | 9~18 days | 2.5~5.1 months | Full-arch | Maxilla | Fixed | Straumann ITI |
| Ma (2010) | 2 weeks& 6 weeks | 3 months | Full-arch | Mandible | Removable | Brånemark,Steri-Oss, Southern,Straumann |
| Payne (2002) | 6 weeks | 3 months | Full-arch | Mandible | Removable | Straumann ITI |
| Røynesdal (2001) | 3 weeks | 3 months | Full-arch | Mandible | Removable | Straumann ITI |
| Tawse-Smith (2002) | 6 weeks | 3 months | Full-arch | Mandible | Removable | Steri-Oss, Southern |
| Turkyilmaz (2007a) | 6 weeks | 3 months | Single | Maxilla | Fixed | Brånemark MKIII |
| Turkyilmaz (2007b) | 1 week | 6 months | Full-arch | Mandible | Removable | Brånemark MKIII |
| Turkyilmaz (2012) | 1 week | 3 months | Full-arch | Mandible | Removable | Brånemark MKIII |

| Table S2. Quality assessment of selected studies | | | | | | | |
|---|---|---|---|---|---|---|---|
| References | Sequence Generation | Allocation Concealment | Blinding (single/double) | Clear definition | Free of selective reporting | Free of other bias | Risk of bias |
| Anil (2012) | NR | NR | NR | Yes | Yes | Yes | High |
| Barewal (2012) | Yes | Yes | Yes | Yes | Yes | Yes | Low |
| De Smet (2007) | No | NR | NR | Yes | Yes | Yes | High |
| Ericsson (2000) | NR | NR | NR | Yes | Yes | Yes | High |
| Engquist (2005) | NR | NR | NR | Yes | Yes | Yes | High |
| Eliasson (2009) | No | No | No | Yes | Yes | No | High |
| Fischer (2008) | Yes | NR | NR | Yes | Yes | Yes | High |
| Tawse-Smith (2002) | Yes | Yes | NR | Yes | Yes | Yes | Moderate |
| Payne (2002) | Yes | Yes | NR | Yes | Yes | Yes | Moderate |
| Turkyilmaz (2012) | Yes | NR | NR | Yes | Yes | Yes | High |
| Turkyilmaz (2007a) | Yes | NR | NR | Yes | Yes | Yes | High |
| Turkyilmaz (2007b) | Yes | NR | NR | Yes | Yes | Yes | High |
| Ma (2010) | Yes | Yes | NR | Yes | Yes | Yes | Moderate |
| R øynesdal (2001) | NR | NR | NR | Yes | Yes | Yes | High |
| NR = not reported. | | | | | | | |
